# Supplementary material for: The TGF-β Receptor Gene Saxophone Influences Larval-Pupal-Adult Development in Tribolium castaneum
Source: Molecules. 2022 Sep 15;27(18):6017. doi: 10.3390/molecules27186017 (PMC9505606; doi:10.3390/molecules27186017)
Supplement: Supplementary file 1 [file molecules-27-06017-s001.zip › molecules-1871577-supplementary.pdf]

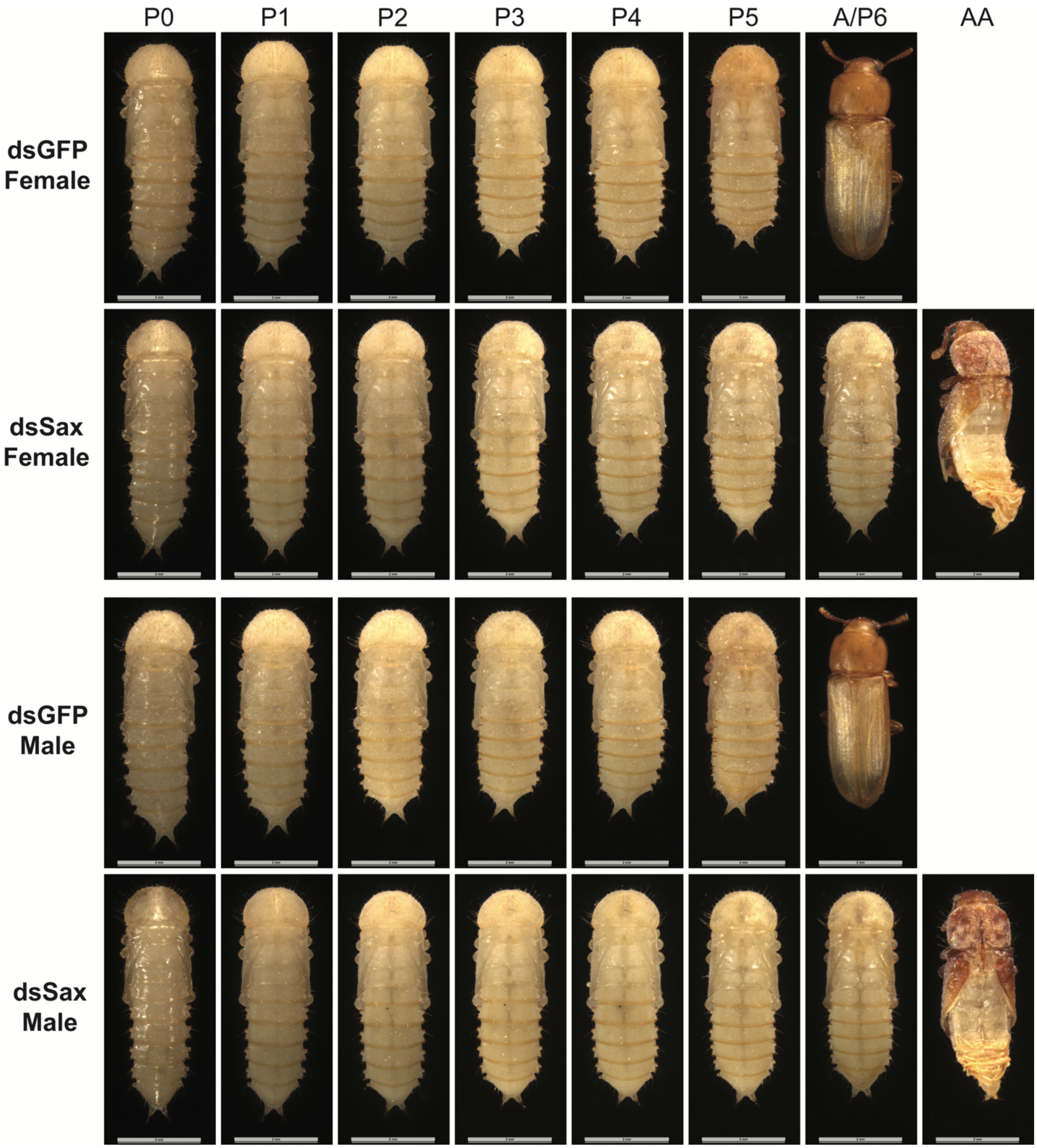

Figure S1. The dorsal view of newly formed female and male pupae and adults from dsGFP (100 ng/larva) and dsSax (100 ng/larva) groups (P0: newly formed pupa; P1: 1-day pupa, P2: 2-day pupa; P3: 3-day pupa; P4: 4-day pupa; P5: 5-day pupa; P6: 6-day pupa; A: adult; AA: abnormal adult).

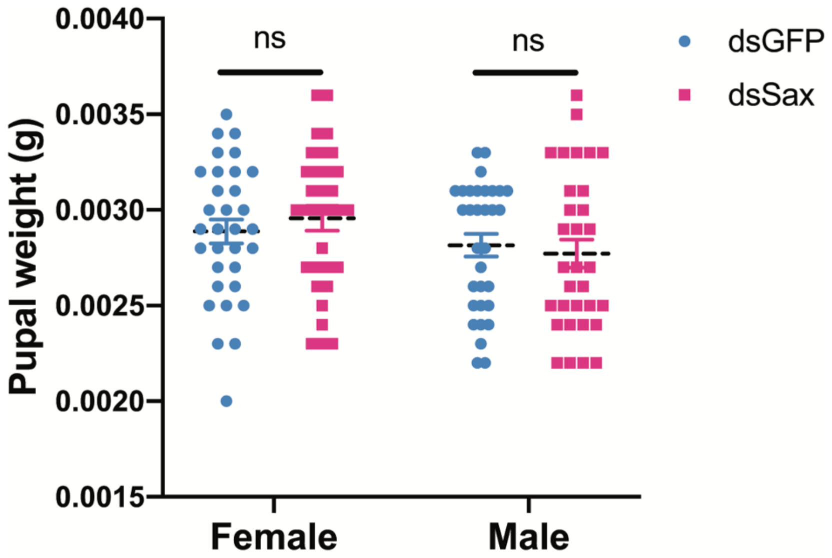

Figure S2. Body weight of female and male pupae from 100 ng/larva dsGFP and dsSax-treated groups. NS indicated non-significant differences between the treatment and corresponding control, $p > 0.05$ by *t*-test.

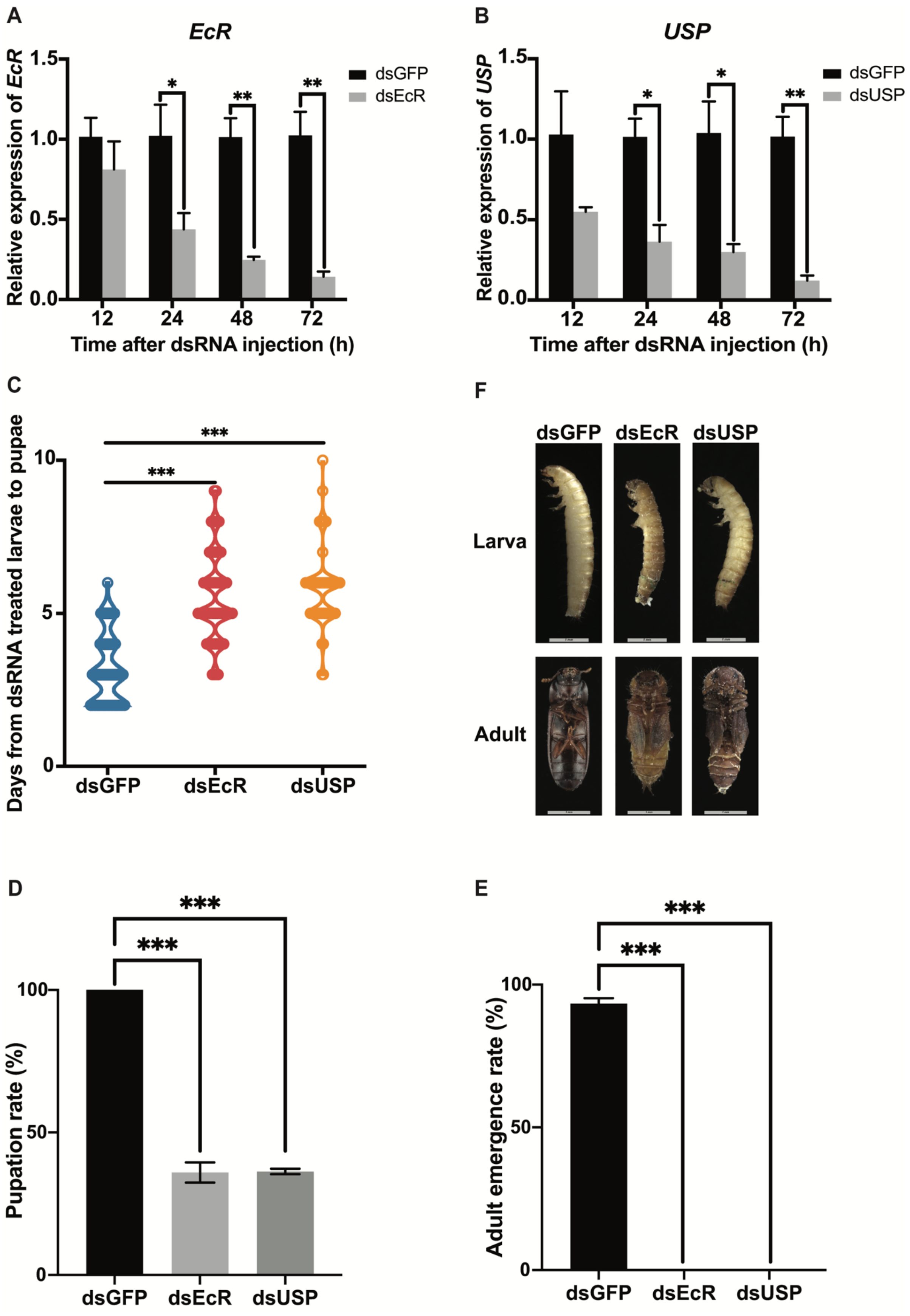
A
EcR
Relative expression of EcR
1.5
1.0
0.5
0.0
dsGFP
dsEcR
*
**
**
12
24
48
72
Time after dsRNA injection (h)
B
USP
Relative expression of USP
1.5
1.0
0.5
0.0
dsGFP
dsUSP
*
*
**
12
24
48
72
Time after dsRNA injection (h)
C
Days from dsRNA treated larvae to pupae
10
5
0
***
***
dsGFP
dsEcR
dsUSP
F
dsGFP
dsEcR
dsUSP
Larva
Adult
D
Pupation rate (%)
100
50
0
***
***
dsGFP
dsEcR
dsUSP
E
Adult emergence rate (%)
100
50
0
***
***
dsGFP
dsEcR
dsUSP

Figure S3. The efficiency of EcR or USP RNAi and their effects on the larval-pupal-adult development of *T. castaneum*. **(A)** Relative expression levels of *EcR* 12-72 h post 100 ng/larva of dsEcR injection. **(B)** Relative expression levels of *USP* 12-72 h post 100 ng/larva of dsUSP injection. **(C)** Days from dsRNA treated 19-day larvae to newly formed pupae from the 100 ng/larva dsGFP, dsEcR and dsUSP-treated groups. **(D)** Pupation rate in the 100 ng/larva dsGFP, dsEcR and dsUSP-treated groups. **(E)** Adult eclosion rate in the 100 ng/larva dsGFP, dsEcR and dsUSP-treated groups. **(F)** The phenotype of larvae or failure pupation larvae and adults or failed emergence of adults from the 100 ng/larva dsGFP, dsEcR and dsUSP-treated groups. Asterisks above bars indicate significant differences between the treatment and corresponding control, *$p < 0.05$, **$p < 0.01$, *** $p < 0.001$ by *t*-test.

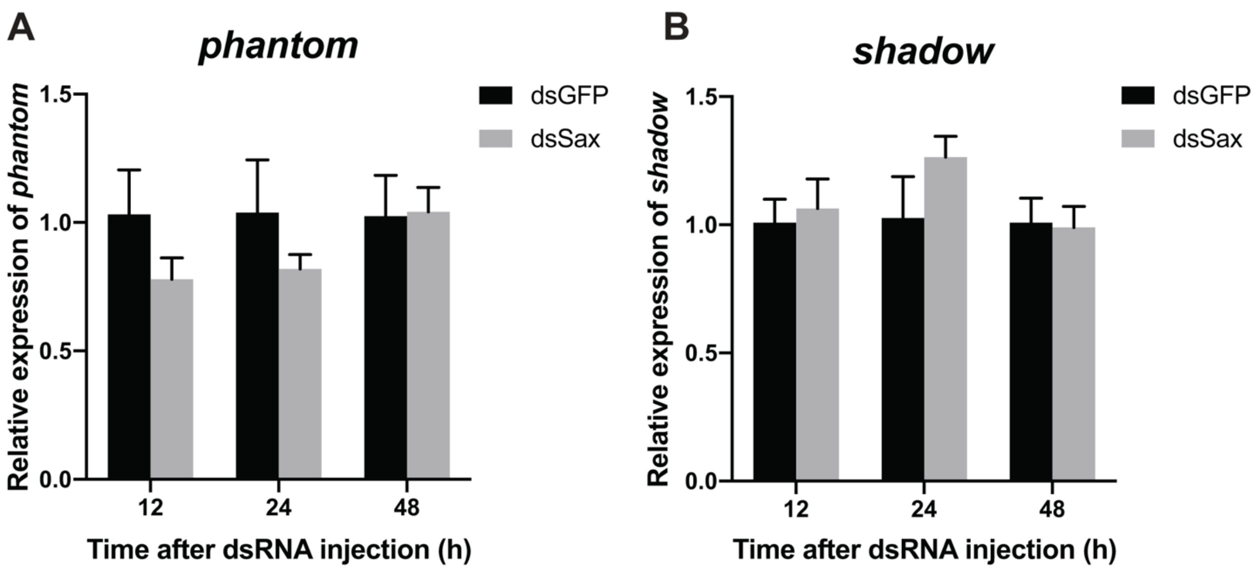

Figure S4. Effects of Sax RNAi on the expression of 20E biosynthesis genes. Relative expression levels of 20E biosynthesis gene *phantom* **(A)** and *shadow* **(B)** 12, 24 and 48 h post dsGFP (100 ng/larva) or dsSax (100 ng/larva) injection.

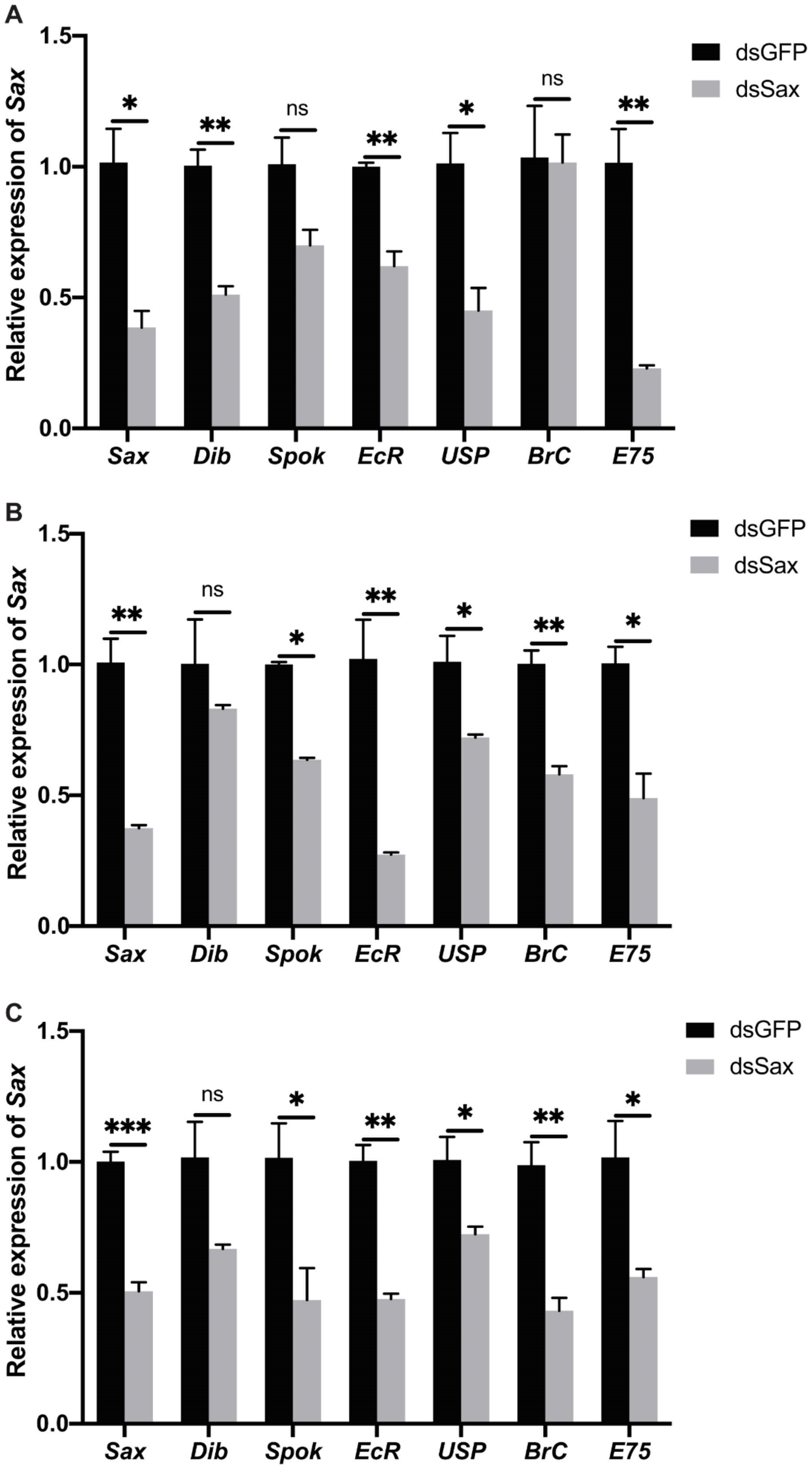
A
B
C
Relative expression of Sax
1.5
1.0
0.5
0.0
dsGFP
dsSax
*
**
***
ns
Sax
Dib
Spok
EcR
USP
BrC
E75

Figure S5. Effects of Sax RNAi on the expression of Sax and 20E pathway-related genes in **(A)** abnormal eyes dissected from dsSax-treated and normal eyes from control larvae, genitalia dissected from 1-day female pupae **(B),** and male pupae **(C)** of dsSax-treated and control larvae. NS indicated non-significant differences between the treatment and corresponding control, $p > 0.05$ by *t*-test. Asterisks above bars indicate significant differences between the treatment and corresponding control, *$p < 0.05$, **$p < 0.01$, *** $p < 0.001$ by *t*-test.

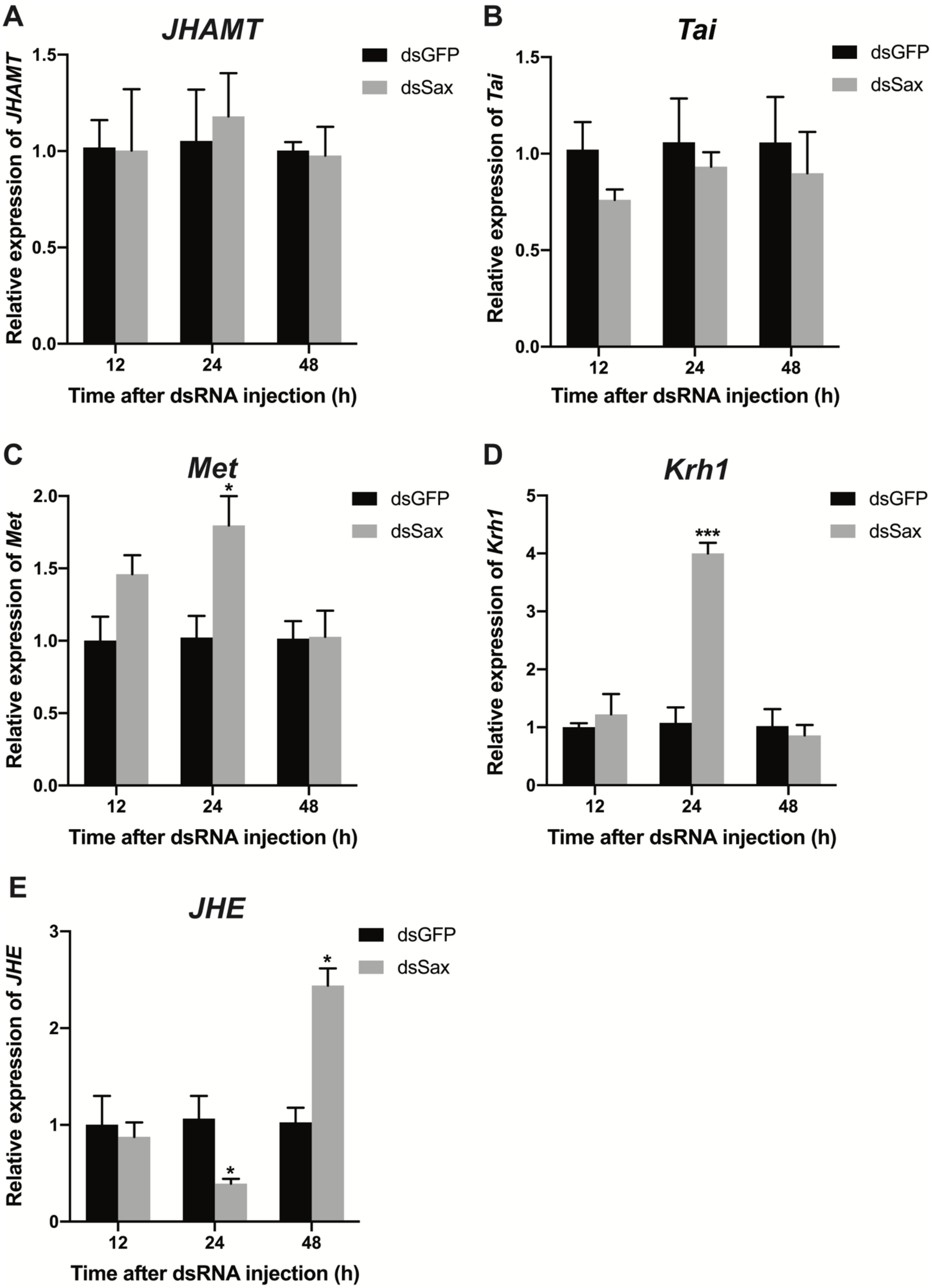

A
JHAMT
Relative expression of JHAMT
1.5
1.0
0.5
0.0
12
24
48
Time after dsRNA injection (h)
dsGFP
dsSax
B
Tai
Relative expression of Tai
1.5
1.0
0.5
0.0
12
24
48
Time after dsRNA injection (h)
dsGFP
dsSax
C
Met
Relative expression of Met
2.0
1.5
1.0
0.5
0.0
*
12
24
48
Time after dsRNA injection (h)
dsGFP
dsSax
D
Krh1
Relative expression of Krh1
5
4
3
2
1
0
***
12
24
48
Time after dsRNA injection (h)
dsGFP
dsSax
E
JHE
Relative expression of JHE
3
2
1
0
*
*
12
24
48
Time after dsRNA injection (h)
dsGFP
dsSax

Figure S6. Effects of Sax RNAi on the expression of JH pathway genes. **(A)** Relative expression levels of JH synthesis related gene *JHAMT* 12, 24 and 48 h post dsGFP (100 ng/larva) or dsSax (100 ng/larva) injection. **(B)** Relative expression levels of JH receptor genes *Tai* and **(C)** *Met* 12, 24 and 48 h post dsGFP (100 ng/larva) or dsSax (100 ng/larva) injection. **(D)** Relative expression levels of JH downstream gene *Krh1* 12, 24 and 48 h post dsGFP (100 ng/larva) or dsSax (100 ng/larva) injection. **(E)** Relative expression levels of JH esterase gene *JHE* 12, 24 and 48 h post dsGFP (100 ng/larva) or dsSax (100 ng/larva) injection. Asterisks above bars indicate significant differences between the treatment and corresponding control, *$p < 0.05$, **$p < 0.01$, *** $p < 0.001$ by *t*-test.

Table S1. Primers used for dsRNA synthesis and qRT-PCR analysis.

Primer name	Sequence (5'-3')	Tm (°C)	Product size (bp)		Purpose
GFP-F	TAATACGACTCACTATAGGGTGGTCCCAATTCTCGTGGAAC	60.3	468		RNAi
GFP-R	TAATACGACTCACTATAGGGCTTGAAGTTGACCTTGATGCC	57.4			
Sax-F	TAATACGACTCACTATAGGGCCACACTCACCCATCAACAG	58.5	465		
Sax-R	TAATACGACTCACTATAGGGGCTGATCCACACACACAACC	59.4			
EcR-F	TAATACGACTCACTATAGGGGTTGAACCAGAATTGTCAGA	53.5	456		
EcR-R	TAATACGACTCACTATAGGGTCTGCAGAAATGCAAGAGAT	55.0			
USP-F	TAATACGACTCACTATAGGGCGAAGGTTGCAAAGGATTTT	55.1	408		
USP-R	TAATACGACTCACTATAGGGGGCAATGATGTGAAATGAGG	55.0			
**Primer name**	**Sequence (5'-3')**	**Tm (°C)**	**Product size (bp)**	**Amplification efficiency (%)**	**Purpose**
RP3-F	TCAAATTGATCGGAGGTTTG	53.9	260	98.5	qRT-PCR
RP3-R	GTCCCACGGCAACATAATCT	58.0			
Sax-F	TGTGACTAGGGGCTGTACGA	60.0	113	101.7	
Sax-R	AGCAATCGACCTGGAACTGG	60.0			
Dib-F	GGATCCTGACGACATTGCCA	60.1	133	97.8	
Dib-R	ATTTGTTGGGAGCAAACCGC	60.0			
Spok-F	CCTGAGCATTTCCTACCGTTC	58.7	90	95.1	
Spok-R	CCCACCCAGAATCCCAAAG	57.7			
phantom-F	AAGAATGTGTGTCGGTGATGAA	58.3	144	94.9	

phantom-R	TCGTGAGGTTTCGGAGTTAGTG	60.0			
shadow-F	TGTCGACTCCGTGGACAATG	60.0	108	102.8	
shadow-R	TGTTCGGCCAGGAGTTTAGG	59.7			
EcR-F	CAGCATAACGAAGAACGCGG	60.0	113	100.1	
EcR-R	CGACGCTGAGGCACTTTTTC	60.1			
USP-F	ACGGAATTGGGTTGTTTGCG	60.0	150	96.1	
USP-R	CCTTCCAGGCTCGTTTGGAT	60.0			
BrC-F	AACGCTGACAGTCTAGCAGG	59.8	150	96.5	
BrC-R	TTGTGGCGTGTAAAAGTGCG	60.0			
E75-F	ACCATCGTGTTCAAGTCCGA	59.3	142	101.1	
E75-R	CGAATTCCAGGTCGGGGATG	60.5			
JHAMT-F	CATCTCGCCCTATCACCATTCG	60.9	149	102.9	
JHAMT-R	CCGCTGAAACCGATTTTGACAA	60.3			
Tai-F	CAGAACGGAACAAAGACTCGG	59.2	128	96.9	
Tai-R	TGTTAATCTGCGCCTGAGGT	59.4			
Met-F	GGGAAAGCAAAGGATCATCA	55.7	152	100.0	
Met-R	AAGGCCTTCTTGCTCACTCA	59.2			
Krh1-F	TGTGACGTTTGCTCGAAGAC	58.8	171	100.5	
Krh1-R	GCACGAGTAGGGCTTTTCAC	59.2			
JHE-F	CGAACCGCTGACTCCGTATTCA	62.6	146	102.2	
JHE-R	CTTCATCTTGGACACTACCCACCATC	63.0			

Table S2. The accession numbers of protein sequences included in the phylogenetic tree.

Taxonomy	Species	Receptor type	Accession number
Chordata	*Labeo rohita*	TGFβRI	KAI2660332.1
	Hypomesus transpacificus	TGFβRI	XP_046882693.1
	Protopterus annecten	TGFβRI	XP_043921044.1
	Xenopus laevis	TGFβRI	XP_041422104.1
	Chlorocebus sabaeus	TGFβRI	XP_037838660.1
	Ophiophagus Hannah	TGFβRI	ETE66478.1
	Mus musculus	TGFβRI	NP_033396.1
	Rattus norvegicus	TGFβRI	NP_036907.2
	Bos taurus	TGFβRI	NP_777046.1
	Sus scrofa	TGFβRI	NP_001033728.1
	Canis lupus familiaris	TGFβRI	XP_038538190.1
	Equus caballus	TGFβRI	XP_023485508.1
	Felis catus	TGFβRI	XP_023098267.2
	Saimiri boliviensis boliviensis	TGFβRI	XP_039331037.1
	Gorilla gorilla gorilla	TGFβRI	XP_030870590.1
	Homo sapiens	TGFβRI	NP_004603.1
	Chelonia mydas	ActivinRI	EMP38745.1
Arthropoda Crustacea	*Penaeus vannamei*	Sax	ROT73998.1
	Homarus americanus	ActivinRI	KAG7155902.1
	Hyalella Azteca	ActivinRI	XP_047735812.1
	Pollicipes pollicipes	ActivinRI	XP_037085320.1
	Chionoecetes opilio	ActivinRI	KAG0715362.1
	Lepeophtheirus salmonis	Sax	CDW20669.1
	Daphnia magna	ActivinRI	XP_045031710.1
	Daphnia pulex	Sax	EFX65169.1
Arthropoda Arachnida	*Argiope bruennichi*	ActivinRI	KAF8792154.1
	Ixodes Ricinus	Sax	JAB69979.1
	Ixodes scapularis	ActivinRI	XP_029835439.2
Arthropoda Insecta Hemiptera	*Panstrongylus megistus*	ActivinRI	JAC87045.1
	Rhodnius neglectus	ActivinRI	JAI52457.1
	Homalodisca vitripennis	ActivinRI	KAG8278389.1
	Halyomorpha halys	ActivinRI	XP_014282404.1
	Cimex lectularius	ActivinRI	XP_014257137.1
	Lygus hesperus	ActivinRI	JAQ17797.1
	Nilaparvata lugens	ActivinRI	XP_039298943.1
	Diaphorina citri	ActivinRI	XP_008467676.1
Arthropoda Insecta Lepidoptera	*Helicoverpa armigera*	TGFβRI	ASK12089.1
	Manduca sexta	ActivinRI	XP_037301442.1
	Operophtera brumata	Sax	KOB71599.1

	Leguminivora glycinivorella	ActivinRI	XP_048000927.1
	Plutella xylostella	ActivinRI	XP_048484336.1
	Aricia agestis	ActivinRI	XP_041980030.1
	Colias croceus	ActivinRI	XP_045505182.1
	Pieris brassicae	ActivinRI	XP_045512665.1
	Pieris napi	ActivinRI	XP_047506282.1
	Papilio machaon	ActivinRI	XP_014365430.1
	Helicoverpa zea	ActivinRI	XP_047035342.1
	Danaus plexippus plexippus	Sax	OWR50038.1
	Melitaea cinxia	ActivinRI	XP_045455937.1
	Maniola jurtina	ActivinRI	XP_045762526.1
	Pararge aegeria	ActivinRI	XP_039758894.1
	Vanessa atalanta	ActivinRI	XP_047540036.1
	Vanessa cardui	ActivinRI	XP_046973643.1
Arthropoda Insecta Coleoptera	*Dendroctonus ponderosae*	ActivinRI	XP_019773630.1
	Sitophilus oryzae	ActivinRI	XP_030762424.1
	Coccinella septempunctata	ActivinRI	XP_044744263.1
	Harmonia axyridis	ActivinRI	XP_045465809.1
	Onthophagus taurus	ActivinRI	XP_022910754.1
	Agrilus planipennis	ActivinRI	XP_018327584.1
	Photinus pyralis	ActivinRI	XP_031354576.1
	Asbolus verrucosus	ActivinRI	RZC40167.1
	Tribolium castaneum	Sax	EFA07576.2
	Tribolium madens	ActivinRI	XP_044264038.1
	Anoplophora glabripennis	ActivinRI	XP_018563389.1
	Diabrotica virgifera virgifera	ActivinRI	XP_028138601.1
	Leptinotarsa decemlineata	ActivinRI	XP_023027570.1
Arthropoda Insecta Neuroptera	*Chrysoperla carnea*	ActivinRI	XP_044727407.1
Arthropoda Insecta Thysanoptera	*Frankliniella occidentalis*	ActivinRI	XP_026280937.1
	Thrips palmi	ActivinRI	XP_034256953.1
Arthropoda Insecta Odonata	*Ischnura elegans*	ActivinRI	XP_046386432.1
Arthropoda Insecta Orthoptera	*Schistocerca americana*	ActivinRI	XP_047001594.1
	Schistocerca piceifrons	ActivinRI	XP_047119584.1
Arthropoda	*Cryptotermes secundus*	ActivinRI	XP_023718523.1

Insecta<br>Blattodea	*Blattella germanica*	ActivinRI	PSN51061.1
	Zootermopsis nevadensis	ActivinRI	KDR18456.1
Arthropoda<br>Insecta<br>Siphonaptera	*Ctenocephalides felis*	ActivinRI	XP_026467222.1
Arthropoda<br>Insecta<br>Diptera	*Megaselia abdit*	Sax	AFK24735.1
	Anopheles gambiae	Sax	AAT07313.1
	Aedes aegypti	ActivinRI	XP_021696047.1
	Culex tarsalis	ActivinRI	JAV32159.1
	Drosophila grimshawi	ActivinRI	XP_001986820.2
	Drosophila mojavensis	ActivinRI	XP_032586684.1
	Drosophila bipectinata	ActivinRI	XP_017097449.2
	Drosophila obscura	ActivinRI	XP_022226804.2
	Drosophila melanogaster	Sax	NP_523652.2
	Drosophila rhopaloa	ActivinRI	XP_016990488.1
	Drosophila takahashii	ActivinRI	XP_017012510.2
	Drosophila biarmipes	ActivinRI	XP_016968226.1
	Drosophila suzukii	ActivinRI	XP_016929999.1
Arthropoda<br>Insecta<br>Hymenoptera	*Nasonia vitripennis*	ActivinRI	XP_031786933.1
	Aphidius gifuensis	ActivinRI	XP_044006153.1
	Athalia rosae	Sax	BBD17789.1
	Diprion similis	ActivinRI	XP_046739434.1
	Neodiprion fabricii	ActivinRI	XP_046474108.1
	Neodiprion virginiana	ActivinRI	XP_046608617.1
	Neodiprion lecontei	ActivinRI	XP_015520402.1
	Neodiprion pinetum	ActivinRI	XP_046474108.1
	Monomorium pharaonis	ActivinRI	XP_036147163.1
	Belonocnema kinseyi	ActivinRI	XP_033224717.1
	Cotesia glomerata	ActivinRI	XP_044587432.1
	Venturia canescens	ActivinRI	XP_043278576.1
	Polistes fuscatus	ActivinRI	XP_043503280.1
	Vespula pensylvanica	ActivinRI	XP_043672158.1
	Vespa crabro	ActivinRI	XP_046823150.1
	Vespa velutina	ActivinRI	XP_047356093.1
	Osmia bicornis bicornis	ActivinRI	XP_029037793.1
	Colletes gigas	ActivinRI	XP_043264007.1
	Apis laboriosa	ActivinRI	XP_043793781.1
	Apis mellifera carnica	ActivinRI	KAG9429780.1
	Bombus pyrosoma	ActivinRI	XP_043598552.1
	Frieseomelitta varia	ActivinRI	XP_043514985.1
